# Supplementary material for: Comparative multiomics analysis of cell physiological state after culture in a basket bioreactor
Source: Sci Rep. 2022 Nov 23;12:20161. doi: 10.1038/s41598-022-24687-4 (PMC9686226; doi:10.1038/s41598-022-24687-4)

11982816:+@NW_023666070.1:11986008:11986103:+@NW_023666070.1:11988586:11988657:+@NW_0236660

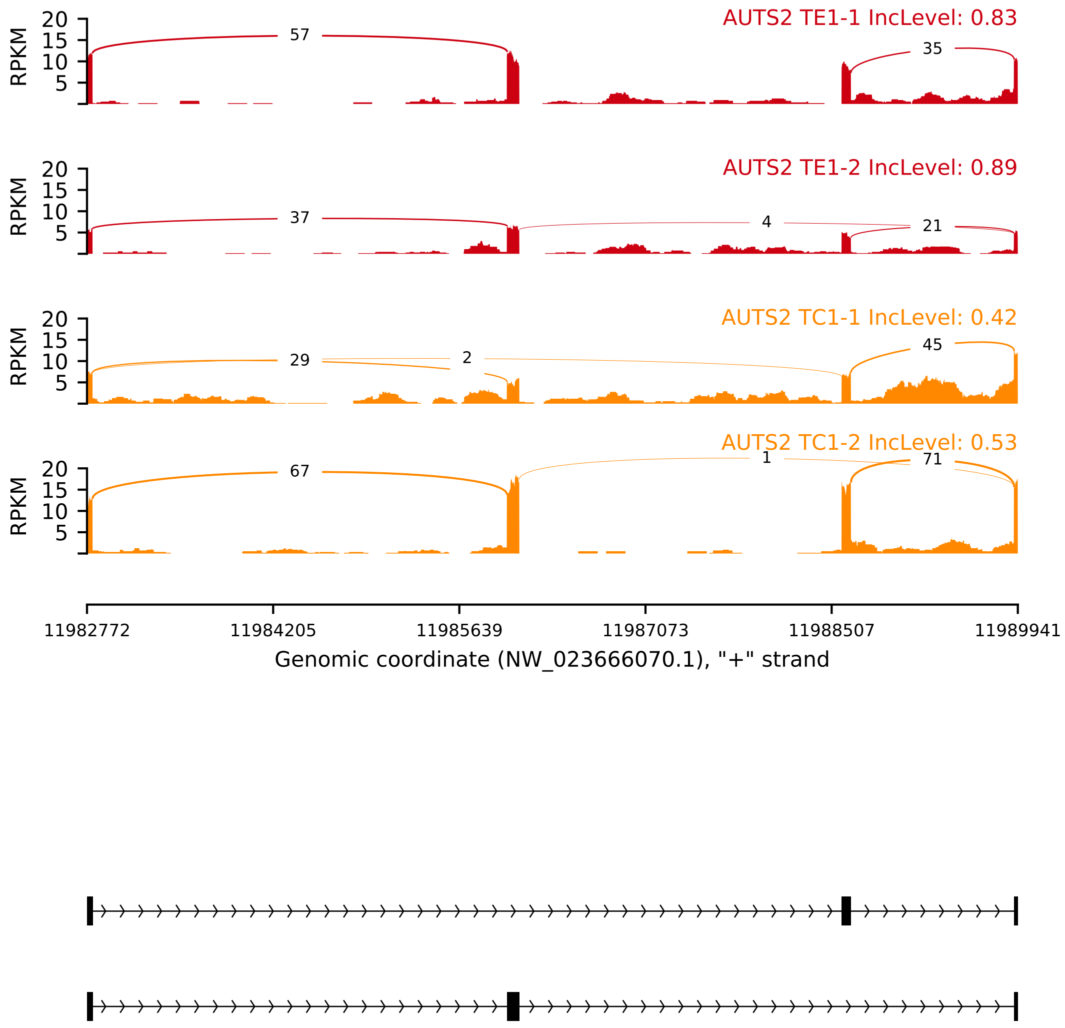

Supplement: Supplementary file 1 — Supplementary Information 1. [file 41598_2022_24687_MOESM1_ESM.zip › raw data/Transcriptomic raw data/7.AS/2.dasplot/TE1vsTC1/MXE/13_AUTS2_NW_023666070.1_11982772_11982816_+@NW_023666070.1_11986008_11986103_+@NW_023666070.1_11988586_11988657_+@NW_023666070.1_11989913_11989942_+.pdf]
